# Epidemiology of Major Non-communicable Diseases in Ethiopia: A Systematic Review

**Published:** 2014-03

**Authors:** Awoke Misganaw, Damen Haile Mariam, Ahmed Ali, Tekebash Araya

**Affiliations:** College of Health Sciences, Addis Ababa University, Addis Ababa, Ethiopia

**Keywords:** Cancer, Cardiovascular disease, Chronic obstructive pulmonary disease, Diabetes, Risk factors, Ethiopia

## Abstract

Impact of non-communicable diseases is not well-documented in Ethiopia. We aimed to document the prevalence and mortality associated with four major non-communicable diseases in Ethiopia: cardiovascular disease, cancer, diabetes, and chronic obstructive pulmonary disease. Associated risk factors: hypertension, tobacco-use, harmful use of alcohol, overweight/obesity, and *khat*-chewing were also studied. Systematic review of peer-reviewed and grey literature between 1960 and 2011 was done using PubMed search engines and local libraries to identify prevalence studies on the four diseases. In total, 32 studies were found, and half of these studies were from Addis Ababa. Two hospital-based studies reviewed the prevalence of cardiovascular disease and found a prevalence of 7.2% and 24%; a hospital-based study reviewed cancer prevalence and found a prevalence of 0.3%; two hospital-based studies reviewed diabetes prevalence and found a prevalence of 0.5% and 1.2%; and two hospital-based studies reviewed prevalence of asthma and found a prevalence of 1% and 3.5%. Few community-based studies were done on the prevalence of diabetes and chronic pulmonary obstructive disease among the population. Several studies reviewed the impact of these diseases on mortality: cardiovascular disease accounts for 24% of deaths in Addis Ababa, cancer causes 10% of deaths in the urban settings and 2% deaths in rural setting, and diabetes causes 5% and chronic obstructive pulmonary disease causes 3% of deaths. Several studies reviewed the impact of these diseases on hospital admissions: cardiovascular disease accounts for 3%-12.6% and found to have increased between 1970s and 2000s; cancer accounts for 1.1%-2.8%, diabetes accounts for 0.5%-1.2%, and chronic obstructive diseases account for 2.7%-4.3% of morbidity. Overall, the major non-communicable diseases and related risk factors are highly prevalent, and evidence-based interventions should be designed.

## INTRODUCTION

Non-communicable diseases are the leading causes of death globally, killing more people each year than all other causes combined. Contrary to popular opinion, available data demonstrate that nearly 80% of deaths due to non-communicable diseases occur in low- and middle-income countries (1). Of the 57 million deaths that occurred globally in 2008, thirty-six million were due to non-communicable diseases comprising mainly cardiovascular diseases, cancers, diabetes, and chronic lung diseases. The combined burden of these diseases is rising fastest among the lower-income countries, populations, and communities (2).

World Health Organization (WHO) estimated in 2011 that 34% of Ethiopian population is dying from non-communicable diseases, with a national cardiovascular disease prevalence of 15%, cancer and chronic obstructive pulmonary disease prevalence of 4% each, and diabetes mellitus prevalence of 2%. Communicable maternal, perinatal and nutritional conditions accounted for 57% of the deaths. This WHO estimation is comparable with East African countries, such as Kenya, Uganda, and Eritrea (3). The resulting double burden of non-communicable diseases, with higher prevalence of pre-existing communicable, maternal, perinatal and nutritional conditions, constrains the already-meagre health resources and hinders economic development in Ethiopia (4) .

Similarly, Global Burden of Disease (GBD) studies estimated age-standardized death rates of 800 per 100,000 population for non-communicable diseases in Ethiopia, of which higher death rates (approximately 450 per 100,000) were attributed to cardiovascular disease and diabetes, 150 per 100,000 attributed to cancer, and 100 per 100,000 to chronic obstructive pulmonary disease (5). These estimations were much higher than in many developed countries. Although these estimates of cardiovascular disease, cancer, diabetes mellitus, and chronic obstructive pulmonary disease look higher in Ethiopia, estimations by WHO and GBD studies are highly uncertain because the causes of deaths were predicted using cause-of-death models due to lack of information on the level of mortality or cause of death at the country level, which should be substantiated by national evidences (6).

Despite the above estimations for global prevalence of the four major non-communicable diseases, cardiovascular disease, cancer, diabetes mellitus, and chronic obstructive pulmonary disease were not well-documented in Ethiopia. On the other hand, accurate information on the prevalence of major public-health importance is required to have informed health policy decision (7,8). Therefore, it is crucial to document prevalence estimations for the major non-communicable diseases for the purposes of research and interventions. We reviewed published and grey literature aiming to document the prevalence and mortality associated with the four major non-communicable diseases in Ethiopia: cardiovascular disease, cancer, diabetes, and chronic obstructive pulmonary disease and the associated risk factors, such as hypertension, tobacco-use, harmful use of alcohol, overweight/obesity, and *khat*-chewing.

## MATERIALS AND METHODS

### Search strategy

A systematic review of peer-reviewed and grey literature was undertaken to identify studies that estimated the prevalence of cardiovascular diseases, cancer, diabetes mellitus, and chronic obstructive pulmonary disease in Ethiopia between 1960 and 2011. We used MeSH of PubMed search engines, using the medical subject titles ‘cardiovascular diseases’, ‘stroke’, ‘hypertension’, ‘myocardial infarction, ‘heart disease’, ‘diabetes mellitus’, ‘neoplasm’, ‘cancer’, ‘asthma’, ‘burden of disease’, ‘non communicable diseases’, combined with the term ‘smoking’, ‘tobacco’, ‘alcohol’, ‘*khat* chewing’, ‘risk factors’, ‘physical exercise’, ‘diet’, and ‘Ethiopia’. The references of included articles were scanned to identify additional articles of interest and used websites of the HINARI and Google Scholar, World Bank, and World Health Organization to access articles. Grey literature was searched from Addis Ababa and Jimma University Libraries and Ethiopian Federal Ministry of Health (Figure).

### Selection of studies

The inclusion criteria used were: (i) articles with clear objectives and methodologies; (ii) articles published from 1960 to 2011; (iii) articles addressing one or more of the four major non-communicable diseases (prevalence of cardiovascular diseases, cancer, diabetes mellitus and chronic obstructive pulmonary diseases); (iv) articles published in English language; and (v) articles for which full texts were obtained for this review (Figure).

### Data extraction

We developed a draft data extraction checklist and piloted it on 10 randomly-selected journals. The checklist was revised and further tested on another randomly-sampled 10 journals, and further refinements were made. In the checklist, information was included on title, author, year of publication, year of data collection, study design, study setting (hospital or community, urban/rural, or mixed), region, population, sample-size and sampling procedure, data-collection procedures, mean age of the study participants, percentage prevalence of cardiovascular diseases/cancer/diabetes/chronic pulmonary diseases (or number of cases), diagnostic criteria, percentage of smokers/alcohol-users/*khat*-chewers/hypertensive patients.

## RESULTS

In total, 32 studies were found to meet the inclusion criteria. Almost half of the studies were from Addis Ababa, the capital city of Ethiopia. Fifteen studies were on cardiovascular diseases, 11 each were on cancer and diabetes mellitus, and 9 were on chronic obstructive pulmonary disease (Figure). In this review, community- and hospital-based studies were used for indicating population prevalence, and mortality and hospitalization studies were used for showing severities of the diseases.

### Cardiovascular diseases

#### Population prevalence

*Community-based studies*: We did not find studies on population prevalence of cardiovascular diseases (Table 1).

**Figure.**
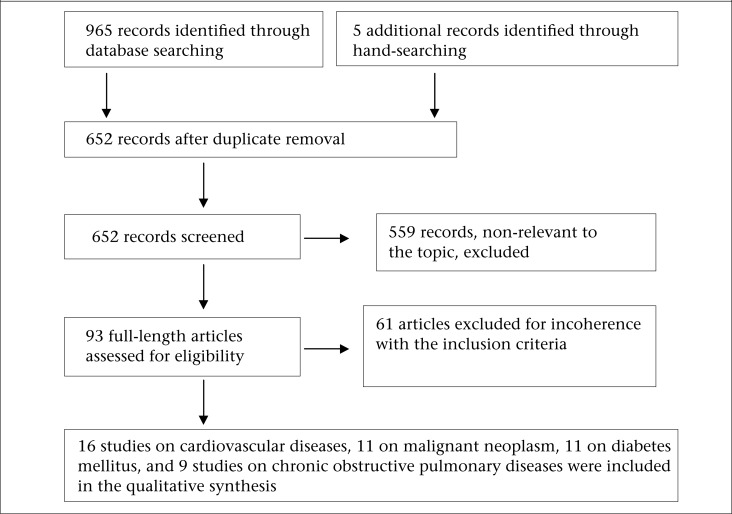
Summary of literature search

*Hospital-based studies*: Five hospital-based studies on the prevalence of cardiovascular diseases and their subtypes were found (Table 1). In a study of sampled patients conducted in the eastern part of the country, an estimated prevalence of 7.2% for cardiovascular diseases and 2.4% for hypertensive heart disease was documented among all age-groups (9). In contrast, a study in the capital city estimated 24% prevalence of cardiovascular diseases among the sampled outpatient visits by adults (10). In a rural hospital study with sampled outpatient visits, an estimated 0.5% hypertension prevalence was found among adults aged 15 years and above (11). In the fourth and fifth studies conducted in the capital city, hypertensive heart disease prevalence was estimated to be 12% among adolescents and adults aged 11 years and above (12) and 6.7% among older adults (13) (Table 1).

#### Severity of the disease

*Mortality*: Three studies investigated mortality among patients with cardiovascular diseases: two population-based studies with the verbal autopsy technique and one hospital-based mortality study. In the first study, with randomly-sampled adult deaths in the capital city, 24% of deaths were attributed to cardiovascular diseases (14) and, in a second study of sampled deaths in Amhara region, 6.5% of deaths were attributed to cardiovascular diseases among all age-groups (15). Congestive heart failure was reported to have caused 2.5% of deaths among all age-groups in the third sampled hospital-based mortality study (16) (Table 1).

*Hospitalization*: Fourteen studies investigated hospitalization of patients with cardiovascular diseases. The hospitalization differs considerably by age, region, and subtypes of cardiovascular diseases. In a study from Addis Ababa, the highest hospitalization was reported (31% of admissions in a hospital for patients aged 60 years and above) (17). Hospitalization of patients with all categories of cardiovascular diseases ranged from 3% in Amhara to 12.6% in Oromia region (16,18-20). A study among all age-groups for admissions in Medical Intensive Care Unit (MICU) in the capital city reported an 8.8% prevalence of hospitalization for acute myocardial infarction (AMI), and the second study from Oromia region reported 9.8% (21,22). The prevalence of cardiovascular diseases appears to have increased over time among hospitalized patients, with studies in the 1970s reporting prevalence of 4.4% while studies in the 2000s reporting 12.6% (18,23) (Table 1).

**Table 1.** Literature review of the prevalence of cardiovascular diseases from hospital-based studies in Ethiopia, 1962-2006

Year	Author	Region in Ethiopia	Urban/Rural	Targets	Sample-size	Outcome	Prevalence (%)	Diagnostic criteria
2011	Misganaw *et al.* (14)	Addis Ababa	Urban	Community-based (>15 years)	3,709 deaths	Cardiovascular disease	24.0	Verbal autopsy
2006	Melaku Z *et al.* (21)	Addis Ababa	Mixed	All age-groups	3,548 MICU admission	AMI disease	9.8	Physicians’ diagnosis
2006	Andarge B *et al.* (18)	Oromia	Mixed	All age-groups	3,99 all admission	Cardiac disease	12.0	Physicians’ diagnosis
2004	Fantahun M *et al.* (15)	Amhara	Mixed	Community-based (all age-groups)	200 deaths	Cardiovascular diseases	6.5	Verbal autopsy
2001	Mamo Y *et al.* (22)	Oromia	Mixed	All age-groups	2,313 MICU admission	AMI disease	8.8	Physicians’ diagnosis
1995	Hussein K (20)	Oromia	Mixed	>10 years	1,440 all admission	Cardiovascular diseases	10.5	Physicians’ diagnosis
1994	Pauletto P *et al.* (11)	Oromia	Rural	>15 years	5,277 outpatients	Hypertension/ heart disease	0.5	Physicians’ diagnosis
1988	Bahta Y *et al.* (31)	Addis Ababa	Mixed	>10 years	917 MICU admission	Cardiovascular disease	11.7	Physicians’ diagnosis
1988	Bahta Y *et al.* (31)	Addis Ababa	Mixed	>10 years	917 MICU admission	Cerebrovascular accident	8.1	Physicians’ diagnosis
1988	Bahta Y *et al.* (31)	Addis Ababa	Mixed	>10 years	917 MICU admission	Congestive heart failure	5.6	Physicians’ diagnosis
1983	Tekelu B (13)	Addis Ababa	Urban	Adults	2,145 outpatients	Hypertension/ heart disease	6.7	Physicians’ diagnosis
1982	Lester FT (12)	Addis Ababa	Mixed	>60 years	200 medical admission	Cardiovascular disease	31.0	Physicians’ diagnosis
1982	Abraham G (19)	Addis Ababa	Mixed	13-82 years	5,667 medical admissions	Cardiovascular disease	6.6	Physicians’ diagnosis
1976	Habte-Gabr E *et al.* (16)	Amhara	Mixed	All age-groups	3,611 all admissions	Cardiovascular disease	3.0	Physicians’ diagnosis
1976	Habte-Gabr E *et al.* (16)	Amhara	Mixed	All age-groups	238 deaths	Congestive heart failure	2.5	Physicians’ diagnosis
1974	Lainovic D (26)	Addis Ababa	Mixed	>15 years	9,330 medical admissions	Cardiovascular disease	6.0	Physicians’ diagnosis
1973	Lester FT (12)	Addis Ababa	Mixed	>11 years	2,103 outpatients	Hypertension/heart disease	12.0	Physicians’ diagnosis
1971	Teklu B *et al.* (34)	Addis Ababa	Urban	17-64 years	460 outpatients	Cardiovascular disease	24.0	Physicians’ diagnosis
1970	Pavlica D (23)	Addis Ababa	Mixed	>16 years	3,922 medical admissions	Cardiovascular disease	4.4	Physicians’ diagnosis
1970	Pavlica D (23)	Addis Ababa	Mixed	>16 years	3,922 medical admissions	Hypertension/heart disease	2.5	Physicians’ diagnosis
1970	Pavlica D (23)	Addis Ababa	Mixed	>16 years	3,922 medical admissions	Rheumatic valvular disease	0.7	Physicians’ diagnosis
1962	Blahos J *et al.* (9)	Harrar	Mixed	All age-groups	11,170 outpatients	Cardiovascular disease	7.2	Physicians’ diagnosis
1962	Blahos J *et al.* (9)	Harrar	Mixed	All age-groups	11,170 outpatients	Hypertension/heart disease	2.4	Physicians’ diagnosis

AMI=Acute myocardial infarction

MICU=Medical Intensive Care Unit

### Cancer

#### Prevalence in population

*Community-based studies:* We did not find studies on prevalence of cancer in population (Table 2).

*Hospital-based studies:* One study on hospital-based prevalence of cancer was found. In this urban hospital study through physicians’ diagnosis, a prevalence of 0.3% was estimated among outpatient adults aged 20 years and above (24) (Table 2).

#### Severity of the disease

*Mortality:* Four studies investigated mortality for patients with cancer: three population-based studies with the verbal autopsy technique and one hospital-based mortality study. In the first study, with randomly-sampled adult deaths in the capital city, 10% prevalence of mortality was attributed to cancer among deaths of those aged 15 years and above (14). In the second study, with sampled deaths in the Amhara region, 2% prevalence of mortality was attributed to cancer among all age-groups (15). The third study of community-based rural sampled deaths in the Southern region of Ethiopia estimated a cancer mortality prevalence of 1.5% (25). A hospital-based study among patients sampled through physicians’ diagnosis also identified a cancer mortality prevalence of 2.9% in all age-groups (16) (Table 2).

*Hospitalization:* Six studies investigated hospitalization for patients with cancer, and three more reported pathological investigations. The highest hospitalization was reported in a study from Addis Ababa: 14.5% of admissions in a hospital for patients aged 60 years and above (17). Hospitalization of patients with cancer ranged from 1.1% to 2.8% in Addis Ababa (23,26). Hospital-based pathological studies estimated cancer prevalence ranging from 8.3% to 27.9% (27,28) (Table 2).

### Diabetes mellitus

#### Population prevalence

*Community-based studies:* Two community-based studies on population prevalence of diabetes were found. A study with urban and rural sampled population in the Southern region estimated the prevalence of diabetes mellitus (type 1 and 2) to be 4.9% among adults aged 18 years and above (29). The second study, with urban sampled population in the Oromia region, estimated the prevalence of type 2 diabetes mellitus to be 5.3% among adults aged 40 years and above (30) (Table 3).

**Table 2.** Literature review of the prevalence of malignant neoplasm in Ethiopia, 1970-2011

Year	Author	Region in Ethiopia	Urban/Rural	Targets	Sample-size	Outcome	Prevalence (%)	Diagnostic criteria
2011	Misganaw A *et al.* (14)	Addis Ababa	Urban	Community-based (>15 years)	3,709 deaths	Malignant neoplasm disease	10	Verbal autopsy
2004	Bezabih M (27)	Oromia	Mixed	Hospital-based (all age-groups)	3,200 specimens	Malignant neoplasm disease	8.3	Pathological
2001	Fantahun M *et al.* (15)	Amhara	Mixed	Community-based (all age-groups)	200 deaths	Malignant neoplasm disease	2	Verbal autopsy
1990	Shamebo M (36)	Addis Ababa	Mixed	Hospital-based 14-80 years	7,969 medical admissions	Leukaemia	2.3	Physicians’ diagnosis
1998	Abdulahi H *et al.* (25)	SNNPR	Mixed	Community-based (all age-groups)	875 deaths	Malignant neoplasm disease	1.5	Verbal autopsy
1986	Aseffa A *et al.* (28)	Amhara	Mixed	Hospital-based (all age-groups)	1,668 specimens	Neoplastic disease	27.9	Pathological
1986	Aseffa A *et al.* (28)	Amhara	Mixed	Hospital-based (all age-groups)	1,668 specimens	Malignant neoplasm disease	14.6	Pathological
1982	Tekelu B (24)	Addis Ababa	Urban	>20 years	2,854 outpatients	Malignant neoplasm	0.3	Physicians’ diagnosis
1982	Lester FT (17)	Addis Ababa	Mixed	Hospital-based (>60 years)	200 medical admissions	Neoplasm disease	14.5	Physicians’ diagnosis
1976	Habte-Gabr E *et al.* (16)	Amhara	Mixed	Hospital-based	3,611 all admissions	Neoplasm disease	2	Physicians’ diagnosis
1976	Habte-Gabr E *et al.* (16)	Amhara	Mixed	Hospital-based (all age-groups)	238 deaths	Hematoma mortality	2.9	Physicians’ diagnosis
1974	Lainovic D (26)	Addis Ababa	Mixed	Hospital-based (>15 years)	9,330 medical admissions	Neoplasm disease	1.1	Physicians’ diagnosis
1970	Pavlica D (23)	Addis Ababa	Mixed	Hospital-based (>16 years)	3,922 medical admissions	Neoplasm disease	2.8	Physicians’ diagnosis
1970	Pavlica D (23)	Addis Ababa	Mixed	Hospital-based (>16 years)	3,922 medical admissions	Primary carcinoma of the liver	2.3	Physicians’ diagnosis

SNNPR=Southern Nations

Nationalities, and Peoples Region

**Table 3.** Literature review of prevalence of diabetes mellitus (DM) in Ethiopia, 1963-2007

Year	Author	Region in Ethiopia	Urban/Rural	Targets	Sample-size	Outcome	Prevalence (%)	Diagnostic criteria
2011	Misganaw A *et al.* [14]	Addis Ababa	Urban	Community-based (>15 years)	3,709 deaths	Diabetes mellitus	5	Verbal autopsy
2011	Giday A *et al.* (29)	SNNPR	Mixed	Community-based (>18 years)	979 sampled population	Diabetes mellitus	4.9	Laboratory tests
2007	Yemane *et al.* (30)	Oromia	Urban	Community-based (>40 years)	576 sampled population	Type 2 diabetes mellitus	5.3	Laboratory tests
2006	Melaku Z *et al.* (21)	Addis Ababa	Mixed	All age-groups	3,548 MICU admissions	Diabetic kitoacidosis	10.7	Physicians’ diagnosis
1988	Bahta Y *et al.* (31)	Addis Ababa	Mixed	>10 years	917 MICU admissions	Diabetic kitoacidosis	9.7	Physicians’ diagnosis
1982	Tekelu B (24)	Addis Ababa	Urban	>20 years	2,854 outpatients	Diabetes mellitus	1.2	Physicians’ diagnosis
1982	Lester FT (17)	Addis Ababa	Mixed	>60 years	200 medical admissions	Diabetes mellitus	11.5	Physicians’ diagnosis
1976	Habte-Gabr E *et al.* (16)	Amhara	Mixed	All age-groups	3,611 medical admissions	Diabetes mellitus	1.7	Physicians’ diagnosis
1976	Habte-Gabr E *et al.* (16)	Amhara	Mixed	All age-groups	238 deaths	Diabetes mellitus	1.3	Physicians’ diagnosis
1974	Lainovic D (26)	Dire Dawa	Mixed	>15 years	9,330 medical admissions	Diabetes mellitus	6	Physicians’ diagnosis
1970	Pavlica D (23)	Addis Ababa	Mixed	>16 years	3,922 medical admissions	Diabetes mellitus	1.8	Physicians’ diagnosis
1963	Blahos J *et al.* (9)	Harrar	Mixed	All age-groups	11,170 outpatients	Diabetes mellitus	0.5	Physicians’ diagnosis

MICU=Medical Intensive Care Unit;

SNNPR=Southern Nations, Nationalities, and Peoples Region

*Hospital-based studies:* Two studies on hospital-based prevalence of diabetes were found. In these studies, the prevalence of diabetes was estimated to be 0.5% in all age-groups and 1.2% among patients aged 20 years and above (9,24) (Table 3).

#### Severity of the disease

*Mortality:* Two studies examined mortality of patients with diabetes. In the first study, with randomly-sampled adult deaths in the capital city, 5% of deaths were attributed to diabetes (14). A hospital-based study with sampled deaths in the Amhara region estimated diabetes-related mortality prevalence of 1.3% in all age-groups (16) (Table 3).

*Hospitalization:* Four studies investigated hospitalization of patients with diabetes, and two more studies investigated one of its complications called diabetic kitoacidosis. The highest hospitalization was reported by a study from Addis Ababa: 11.5 % of admissions in a hospital for patients aged 60 years and above (17). Hospitalization of patients with diabetes ranged from 0.5% in all age-groups to 6% for patients aged 15 years and above (9,26). Studies estimated the prevalence of diabetic kitoacidosis to be 9.7% for patients aged 10 years and above (31) and admitted to Medical Intensive Care Unit (MICU); the figure for all age-groups for the same disease was 10.7% (21) (Table 3).

### Chronic obstructive pulmonary diseases

#### Prevalence in population

*Community-based studies:* One study on population prevalence of chronic obstructive pulmonary disease subtype called ‘asthma’ was found. In this study, which used an urban/rural sampled population and the verbal autopsy technique, 0.6% prevalence was estimated in all age-groups (25) (Table 4).

*Hospital-based studies:* Two studies on hospital-based prevalence of asthma were found. In these studies, the prevalence of asthma was estimated to be 1% and 3.5% among patients aged 20 years and above (24,32) (Table 4).

#### Severity of the disease

*Mortality:* Five community-based studies examined mortality for patients with chronic obstructive pulmonary diseases and asthma, using the verbal autopsy technique. In the first study, with randomly-sampled adult deaths in the capital city, 3% of deaths were attributed to chronic obstructive pulmonary diseases (14). In the second study, which took sampled deaths in the Southern region of Ethiopia, 5.2% of deaths were attributed to chronic obstructive pulmonary diseases among people aged 15-49 years (33) (Table 4). Its subtype—asthma—was estimated to have caused 0.6% of deaths in a sampled community-based study (25).

*Hospitalization:* Two studies investigated hospitalization for patients with chronic obstructive pulmonary diseases. These studies from Addis Ababa estimated 2.7% and 4.3% prevalence of hospitalization for chronic obstructive pulmonary diseases (23,32) (Table 4).

### Prevalence of risk factors of the four non-communicable diseases

Reviewed studies that have been conducted on the major non-communicable diseases since 1984 have mainly addressed the urban population and the adult group (15 years and older). As for risk factors, these studies have dealt with hypertension, higher glucose level (diabetes mellitus), tobacco-use, harmful use of alcohol, being overweight/obese, and *khat-*chewing (Table 5).

In the capital city Addis Ababa, hypertension prevalence ranged from 4.1% among adult workers in 1984 to 30% among a sampled population in 2009 (6,34). In the regions, hypertension prevalence accounted for about 10% in the Southern Nations, Nationalities, and Peoples Region (SNNPR) in 2011 (29), and 1.8% in the rural Amhara populations in the mid-1980s (35) (Table 5).

Similarly, the prevalence of being overweight in the population of Addis Ababa accounted for 25.1% (36) among adult workers in particular and 30.5% (6) of the adult population in general in 2009. One of the regions, viz. SNNPR, accounted for 8.7% of the study population aged 18 years and above (29). The same studies further indicated an obesity prevalence of 5.3% (36) and 7.2% (6) respectively among adult workers in particular and the adult population in general in Addis Ababa (Table 5).

Regarding excessive alcohol-use, the prevalence ranged from 23% to 62% in Addis Ababa (6,36) while the figure for SNNPR was 6.5% (29). On the other hand, current smoking in Addis Ababa was reported to range from 2.2% to 9% (6,36) while the lifetime prevalence of smoking in the SNNPR was reported to be 2.1% (29). A higher *khat*-chewing prevalence of 9.2% was reported from SNNPR (29) and, in Addis Ababa, it ranged from 7.3% to 8.5% (6,36) (Table 5).

**Table 4.** Literature review of the prevalence of chronic obstructive pulmonary diseases in Ethiopia, 1970-2001

Year	Author	Region in Ethiopia	Urban/Rural	Targets	Sample-size	Outcome	Prevalence (%)	Diagnostic criteria
2011	Misganaw A *et al.* (14)	Addis Ababa	Urban	Community based (>15 years)	3,709 deaths	COPD	3	Verbal autopsy
2001	Fantahun M *et al.* (15)	Amhara	Mixed	Community-based (all age-groups)	200 deaths	Asthma	2	Verbal autopsy
2004	Lulu K *et al.* (33)	SNNPR	Mixed	Community-based (15-49 years)	515 deaths	COPD	5.2	Verbal autopsy
1998	Abdulahi H *et al.* (25)	SNNPR	Mixed	Community-based (all age-groups)	875 deaths	Asthma	0.6	Verbal autopsy
1998	Abdulahi H *et al.* (25)	SNNPR	Mixed	Community-based (all age-groups)	575 patients	Asthma	2.3	Algorithm
1982	Tekelu B (24)	Addis Ababa	Urban	>20 years	2,854 outpatients	Asthma	3.5	Physicians’ diagnosis
1977	Lester FT (32)	Addis Ababa	Mixed	>20 years	5,900 medical admissions	Asthma	2.7	Physicians’ diagnosis
1977	Lester FT (32)	Addis Ababa	Mixed	>20 years	26,314 outpatients	Asthma	1	Physicians’ diagnosis
1970	Pavlica D (23)	Addis Ababa	Mixed	>16 years	3,922 medical admissions	Asthma	4.3	Physicians’ diagnosis

COPD=Chronic obstructive pulmonary diseases

SNNPR= Southern Nations, Nationalities, and Peoples Region

**Table 5.** Literature review of the prevalence of NCD risk factors from community based studies in Ethiopia, 1984-2011

Year	Author	Region in Ethiopia	Urban/Rural	Population (age in years)	Sample-size	HTN	Overweight /Obesity	Alcohol-use	Lifetime smoking	Current smoking	*Khat-*chewing
2011	Giday A *et al.* (29)	SNNPR	Mixed	Community-based (>18 years)	979	9.9	8.7/1	6.5	2.1	NA	9.2
2010	Tran A *et al.* (36)	Addis Ababa	Urban	Institution-based adult workers	1,935	17.8	25.1/5.3	23	13	9.0	8.5
2009	Tesfay F *et al.* (6)	Addis Ababa	Urban	Community-based (25-64 years)	3,713	30	30.5/7.2	62	NA	2.2	7.3
1986	Zain A *et al.* (35)	Amhara	Rural	Community-based (>15 years)	478	1.8	NA	NA	NA	NA	NA
1984	Tekelu B (34)	Addis Ababa	Urban	Institution-based adult workers	933	4.1	NA	NA	NA	NA	NA

HTN=Hypertension; NA=Not available

SNNPR=Southern Nations, Nationalities, and Peoples Region

## DISCUSSION

### Main findings

Despite the limitations of our review as we did not conduct quality assessment for studies and potential publication bias with limitation of generalizability, we feel that the published and unpublished data we have presented reflect the comparative sparse data for Ethiopia and future direction for research on non-communicable diseases.

This review indicates that major non-communicable diseases—cardiovascular disease, cancer, diabetes mellitus, and chronic obstructive pulmonary disease—are causing higher proportions of morbidity and mortality, impacting both in the rural and urban populations of Ethiopia. These findings support evidences from sub-Saharan Africa where non-communicable diseases pose a substantial burden (37). The prevalence of certain non-communicable diseases, such as cardiovascular disease, diabetes, cancer, and chronic obstructive pulmonary disease, is increasing rapidly, particularly in the urban areas of sub-Sahara Africa, and that significant demands are being made on the health services by patients with these diseases (37).

Studies also indicated that an epidemiological transition is occurring in Arica, especially in the urban population while people are also hard-hit by HIV/AIDS and tuberculosis (38,39). This increase in non-communicable diseases is expected in the future, especially in relation to ‘Westernization’ of people's diet and lifestyle changes in the urban setting of Africa (38). In our review, the impact of major non-communicable diseases might vary with type of disease, age, and region in Ethiopia. This burden is becoming a big challenge to the healthcare delivery system of the country (4). Increased diagnosis of non-communicable diseases will lead to a corresponding need for greater capacity of the existing health facilities, which are currently over-stretched to diagnose and treat these conditions and also a need for aggressive primary programmes as late diagnosis leads to poor health outcomes (5).

Risk factors of the major non-communicable diseases, such as tobacco-use, excessive alcohol-use, hypertension, being overweight/obese, higher glucose level, and *khat*-chewing, were highly prevalent among the urban population and people aged 15 years and above. According to WHO, non-communicable diseases are caused, to a large extent, by four behavioural risk factors that are pervasive aspects of economic transition, rapid urbanization, and lifestyles of the 21st century: tobacco-use, unhealthy diet, insufficient physical activity, and excessive alcohol-use (40). Estimations indicate a national prevalence of 2.4% current daily tobacco smoking and an adult per-capita consumption of 4.1 litre of pure alcohol in Ethiopia. These behavioural risk factors subsequently lead to four key metabolic/physiological changes: raised blood pressure, raised blood glucose, overweight, and obesity. A national prevalence of raised blood pressure was estimated to be 35.2%, overweight 7.4%, and obesity 1.1% in Ethiopia (3).

### Strengths and weaknesses

In the absence of vital statistics system, epidemiological studies on non-communicable diseases, with a variety of designs and in-depth analysis of risk factors and the effects of interventions, could provide a better understanding of the situations in Ethiopia and provide information to healthcare policy-making. Although this review includes many hospital-based studies which are largely non-representative of the community, it can highlight gaps on the understanding of the major non-communicable diseases in the country. Future research priorities for the country should include better quantification of the major non-communicable diseases and locally-important risk factors. There is a need for comprehensive investigation of population prevalence of cardiovascular diseases, cancer, diabetes mellitus, chronic obstructive pulmonary disease and their risk factors in the country.

### Implications of findings

The prevalence of major non-communicable diseases in Ethiopia is high, with probable underreporting, and will certainly increase in the upcoming years. We believe that proactive thinking is essential in order to mitigate the effects of this hidden or latent epidemic and to provide critical data for formulating evidence-based health policy and interventions. Moreover, primary prevention integrated with the primary healthcare system could be the best way to reduce the burden both in the rural and urban settings of the country. Primary prevention mechanisms, such as increasing awareness and strengthening legislative measures (e.g. tobacco) and health promotion measures, can enhance healthy behaviours and mitigate the rise in the incidence of major non-communicable diseases in the country.

### Conclusions and recommendations

We feel that the published and unpublished data we have presented reflect the comparative sparse data for Ethiopia and future direction for research on major non-communicable diseases despite certain limitations of our review.

Cardiovascular disease, cancer, diabetes mellitus, and chronic obstructive pulmonary disease are highly prevalent and causing higher proportions of morbidity and mortality, impacting both in the rural and urban population of Ethiopia. Their impact varies with type of disease, age, and region. Hospitalization impacts of cardiovascular diseases have increased over time within the last five decades. This burden is becoming a big challenge to the healthcare delivery system of the country. Their risk factors: tobacco-use, harmful use of alcohol, hypertension, overweight/obesity, higher glucose level, and *khat*-chewing were also highly prevalent, mainly in the urban population aged 15 years and above. We believe that proactive thinking is essential in order to mitigate the effects of these hidden or latent epidemics. Therefore, we recommend the following:

- Funding for researchers to conduct large population-based prevalence studies
- Designing population-wide interventions to address the major non-communicable diseases
- Capacity-building of the primary healthcare delivery system to prevent and control the epidemics of non-communicable diseases.

## ACKNOWLEDGEMENTS

We thank Ato Legesse Alemayehu who contributed in the literature search.
